# Caregiver Perceptions and Acceptability of a Provitamin A Carotenoid, Iron and Zinc Rich Complementary Food Blend Prepared from Common Bean and Pumpkin in Rural Uganda

**DOI:** 10.3390/nu12040906

**Published:** 2020-03-26

**Authors:** Edward Buzigi, Kirthee Pillay, Muthulisi Siwela

**Affiliations:** 1 Department of Dietetics and Human Nutrition, School of Agricultural, Earth and Environmental Sciences, University of KwaZulu-Natal, Private Bag X01, Scottsville 3209, Pietermaritzburg 3201, South Africa; pillayk@ukzn.ac.za (K.P.); siwelam@ukzn.ac.za (M.S.); 2 Health Economics and HIV/AIDS Research Division (HEARD), University of KwaZulu-Natal, Westville Campus, J Block 4th Floor, Durban 4041, South Africa; 3 Department of Human Nutrition & Home Economics, Kyambogo University, Kyambogo P.O. Box 1, Uganda

**Keywords:** complementary foods, common bean pumpkin blend, pumpkin blend, provitamin A carotenoids, iron, zinc, caregiver acceptability, Uganda

## Abstract

Ugandan children are vulnerable to vitamin A deficiency (VAD), iron deficiency (ID) and zinc deficiency (ZnD) because they are fed on complementary foods (CFs) low in vitamin A, iron and zinc. This study developed a novel provitamin A carotenoid (PVAC), iron and zinc rich common bean pumpkin blend (BPB) complementary food (CF) from locally available pumpkin and common bean in Uganda and aimed to determine its acceptance, compared to a control pumpkin blend (PB). Seventy caregivers participated in the study. The sensory attributes (taste, colour, aroma, texture and general acceptability) of BPB and PB were rated using a five-point facial hedonic scale (1 = very bad, 2 = bad, 3 = neutral, 4 = good, 5 = very good). Focus group discussions (FGDs) were conducted to assess the perceptions of caregivers about the BPB. The chi square test was used to detect the proportion difference for each sensory attribute between BPB and PB, whilst FGD data were analysed by thematic analysis. A proportion of 64% to 96% of the caregivers rated both BPB and PB as acceptable (good to very good) for all the sensory attributes. There was no significant difference in caregiver acceptability for all attributes between BPB and PB (*p* > 0.05). Caregivers had positive perceptions about the taste, texture, aroma and colour of the BPB. Caregivers were keen to know the specific varieties of common bean and pumpkin used to formulate the PVAC, iron and zinc rich BPB. In conclusion, BPB was acceptable to caregivers, and they were interested to know how to prepare and use it as a CF.

## 1. Introduction

Complementary feeding is defined as a critical period of child growth and development (6 to 24 months of age), when breastmilk should be supplemented with nutrient-rich complementary foods [1,2]. Feeding low quality complementary foods (CFs) during this critical period of growth and development increases the risk of morbidity, mortality and growth faltering [3,4]. 

Micronutrients such as vitamin A, iron and zinc are necessary for child growth, and preventing childhood illnesses such as night blindness, diarrhoea, respiratory tract infections and iron deficiency anaemia [3]. From the age of 6 months, breast milk alone is no longer sufficient to meet the child’s nutritional requirements for vitamin A, iron and zinc [5,6,7]. Therefore, children need to be fed on vitamin A, iron and zinc rich CFs [2]. However, caregivers from developing countries including Uganda feed their children low quality homemade CFs predominantly formulated from staple cereals and tubers such as white maize, sweet potatoes, cassava and yams [8,9,10]. These staple cereals and tubers are rich sources of calories, however, they are low in vitamin A, iron and zinc [11]. Consumption of such staples during the period of complementary feeding is associated with child vitamin A deficiency (VAD), iron deficiency (ID) and zinc deficiency (ZnD) [9].

To combat VAD, ZnD and ID during complementary feeding, it is recommended that children should be fed micronutrient fortified CFs, food supplements and animal source foods (ASFs) [1,2]. This recommendation is plausible because fortified CFs, food supplements and ASFs are rich sources of vitamin A, iron and zinc [12,13,14,15]. However, rural caregivers from developing countries including Uganda lack both physical and economic access to fortified foods, food supplements and animal source foods [16,17]. One potential and sustainable recommended strategy to combat micronutrient deficiencies such as VAD, ID and ZnD is to feed children CFs formulated from intrinsically vitamin A, iron and zinc rich foods, that are locally available, acceptable, and affordable to caregivers [1]. 

In Uganda, common bean and pumpkin are widely cultivated and are affordable to the rural poor [18,19]. Pumpkin is a rich source of provitamin A carotenoids (PVAC), a proformed form of vitamin A [20,21,22]. When pumpkin is consumed by humans, the PVACs are bioconverted into retinol, measured as retinol activity equivalents (RAE), a form of vitamin A used by the human body [23]. Furthermore, common bean is rich in iron and zinc [24,25,26,27]. Cooked pumpkin has over 100% PVAC retention [28,29], whilst common bean has over 90% retention for either iron or zinc retention [24,25]. To this end, by using home-cooking methods, this study prepared a novel PVAC, iron and zinc rich complementary food (CF) blend from locally available common bean (*Obwelu*) and pumpkin (*sweet cream*). 

Testing for child and caregiver sensory acceptability of novel CFs is necessary because the former is the target consumer of CFs, whilst the latter decides on whether to give or not to give the novel CF to the child [30]. It is worth noting that, based on sensory acceptability, caregivers decide whether they will or will not offer the CFs to their children [30,31]. Moreover, testing for sensory acceptability among children is not appropriate because they are too young to make a rational judgment about sensory attributes of CFs [31,32]. Therefore, the aim of this study was to assess the caregiver perceptions and acceptability of a novel common bean pumpkin blend (BPB) compared to a control CF pumpkin blend (PB) in rural Uganda. 

## 2. Materials and Methods 

### 2.1. Study Setting

This study was conducted in rural Kyankwanzi district, central Uganda, an area with a high prevalence of illiteracy and young child caregivers [33]. Children from this socioeconomically disadvantaged area are fed on CFs low in vitamin A, iron and zinc [10,11].

### 2.2. Description of the Homemade Complementary Foods Used in the Study

#### 2.2.1. Ingredients for Preparation of the Complementary Foods

**Figure 1:**
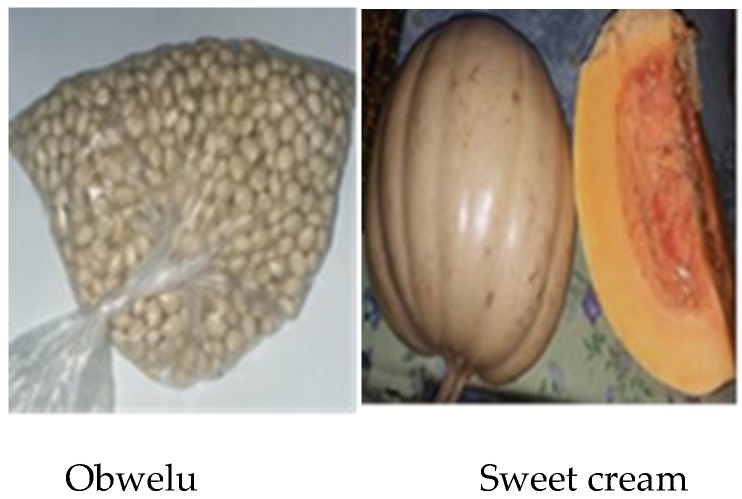
Common bean, *Obwelu* and pumpkin, *Sweet cream* used in the preparation of the complementary foods (CFs).

This study formulated homemade CFs, BPB and PB from common bean (*Obwelu*) and pumpkin (*Sweet cream*). These ingredients were chosen because common bean is rich in iron and zinc [24,25,26,27] and pumpkin is rich in PVAC [20,21,22]. Moreover, these ingredients are cultivated in the study area and available in the local markets [18,19]. Figure 1 shows common bean (*Obwelu*) and pumpkin (*Sweet cream*) used to prepare BPB and PB. 

#### 2.2.2. Preparation of BPB and PB

The Food and Agriculture Organization of the United Nations (FAO) recommends that caregivers should prepare homemade CFs based on the consistency (thinness and thickness) of the food in relation to the child’s age and ability to swallow [1]. To this end, CFs were prepared in accordance with food consistency as recommended by the 2017 FAO guide to conducting participatory cooking demonstrations to improve complementary feeding practices [1]. This 2017 FAO guide encourages participatory cooking demonstrations involving community nutrition and health workers, mother-leaders, and peer counsellors [1]. To this end, the BPB and PB CFs were prepared by child caregivers (expert peer mothers). Community health workers identified 10 expert peer mothers from the local community and invited them to Ntwetwe Health Centre to participate in the preparation of the CFs used in the acceptability study. Expert peer mothers were encouraged to prepare CFs using the locally acceptable homebased methods used in the community to prepare common bean and pumpkin for child consumption. 

Common bean, *Obwelu* and pumpkin, *Sweet cream* were purchased from the local market. Expert peer mothers prepared *Sweet cream* by peeling and discarding seeds followed by boiling (30 to 45 minutes) the pulp. For *Obwelu*, they used overnight soaking (8 to 12 hours) followed by boiling (1 to 1.5 hours). After cooking, caregivers mentioned that they prepare homemade CFs in their community based on consistency (thinness or thickness of the food) suitable for the child’s stage of development. To this end, after cooking by expert peers, research assistants mixed the ingredients to form CFs based on the consistency as recommended by the 2017 Food and Agriculture Organization of the United Nations (FAO) guide to conducting participatory cooking demonstrations to improve complementary feeding practices [1]. Research assistants prepared three different formulations of BPB by mixing and mashing *Sweet cream* and *Obwelu* together. Table 1 shows the ratio of mixing *Sweet cream* and *Obwelu*.

Caregivers, usually mothers are the gatekeepers for CFs [1,34]. Therefore, after preparing, the three formulations of BPB were put on a table in three different serving dishes and presented to the expert peer mothers to select the one that they would choose to feed their children, 6 to 24 months old. Expert peer mothers, one by one entered the room to choose one formulation of BPB. All of the 10 expert mothers unanimously selected BPB-3 prepared by mixing two parts of *Sweet cream* and one part of *Obwelu*. The average age for children of expert peer mothers who selected BPB-3 was 12.1 months. Mashed cooked pumpkin in Uganda is usually given as a single CF [35]. Therefore, a pumpkin blend (PB) prepared from *Sweet cream* was used as a control in this study. Duplicate samples of prepared BPB (test food) and PB (control) were transported to METLAB East Africa Limited Laboratory, Kampala, Uganda for PVAC, iron and zinc analysis.

#### 2.2.3. Vitamin A, Iron and Zinc Content of Formulated Complementary Foods 

PVAC content was analysed by high performance liquid chromatography (HPLC) as described in the HarvestPlus handbook for carotenoid analysis [36]. To analyse the vitamin A content, the Institute of Medicine (2001) bioconversion rates of PVAC to vitamin A, retinol (retinol activity equivalents) were used, i.e., 12µg of ꞵ-carotene is equivalent to 1µg of retinol, whilst 24µg of α-carotene is equivalent to 1µg retinol [37]. Iron and zinc concentrations of CFs were determined by flame atomic absorption spectroscopy (FAAS) as described elsewhere [11,38]. Triplicate analysis for each CF was conducted to get an average of each micronutrient in each CF. 

The Recommended Dietary Allowance (RDA) is the intake that meets the nutrient need of almost all (97% to 98%) individuals in a group [37]. The RDA for vitamin A, iron and zinc for a child 12 to 24 months old is 300 µg RAE/day, 7 mg/day and 3 mg/day, respectively [37]. However, the World Health Organization (WHO) and FAO recommend that a CF should meet at least 50% of the RDA. Table 2 shows the PVAC, vitamin A, iron and zinc content of BPB and PB per 100g of edible portion. Furthermore, it shows the expected 50% iron, zinc and vitamin A content of 100g of a standard complementary food (SCF) for children 12 to 24 months old based on 2013 WHO/FAO Guidelines on formulated CFs for older infants and young children [39]. 

### 2.3. Sensory Evaluation

A pilot study of the sensory evaluation and the focus group discussion was conducted two days prior to the main study. Ten caregivers with children aged 6 to 24 months attending the young child clinic (YCC) for child immunisation and growth monitoring at Ntwetwe Health Centre IV, Kyankwanzi district, participated in a sensory evaluation pilot study. A short while later, all the caregivers who had participated in the sensory evaluation, participated in a pilot focus group discussion (FGD). The aim of the pilot study was to test the sensory evaluation questionnaire, procedures for conducting the sensory evaluation and the focus group discussions (FGDs). The pilot study was conducted on a different day to the main study in order to prevent the pilot study participants from participating in the main study. As the pilot study venue was too far away from the YCC, a closer alternative venue was used for the main study. No changes were made to the sensory evaluation questionnaire after the pilot study.

#### Procedure for Sensory Evaluation

A sample size of more than 50 caregivers is adequate for a valid sensory acceptability study [40]. Accordingly, 70 caregivers, who cared for children aged 6 to 24 months at the time of the study, participated in the sensory evaluation study. Sensory evaluation was conducted in a designated private room, close to the YCC. The caregivers were seated a distance away from each other and were asked not to communicate with each other during the sensory evaluation session. Samples were labelled with random numbers so that panellists would not form judgments based on labels, but rather on their sensory experiences [41]. To this end, the samples were randomly labelled, using a unique three-digit code obtained from a table of random numbers, and were served in a random order [41].

The CF samples were warmed in a microwave oven for 10 seconds, at a medium heat, before serving. Each caregiver received 100g samples of BPB and PB in separate polystyrene cups. The caregivers were provided with a spoon and a cup of water to rinse their palates between samples. A facial hedonic scale has been found to be appropriate for use by semi- illiterate and illiterate panellists [40]. Therefore, caregivers rated the taste, texture, aroma, colour and overall acceptability of both BPB and PB (control) samples using a five-point facial hedonic scale (1 = very bad, 2 = bad, 3 = neutral, 4 = good and 5 = very good) based on the sensory evaluation questionnaire developed in the local language (Luganda). Research assistants explained the questionnaire to the caregiver panellists and assisted them during the evaluation when necessary. 

### 2.4. Focus Group Discussions 

Focus group discussions (FGDs) were conducted to assess caregivers’ acceptability or willingness to use the BPB as a CF. FGDs were conducted about 30 minutes after the sensory evaluation study was completed. All the 70 caregivers who participated in the sensory evaluation were willing to participate in the FGDs. The acceptable sample size for a FGD is between 7 and 12 participants [42]. To this end, participants were divided into 7 focus groups, each containing 10 participants. The study sample had 7 male caregivers. Therefore, it was ensured that at least 1 male caregiver was allocated to each of the 7 FGDs to ensure male representation in each of the focus groups. 

Through established community relationships, four facilitators, experienced in conducting FGDS in the local language (Luganda) were recruited for a one-day training in focus group moderation in relation to the specifics of this research study following guidelines explained elsewhere [43]. A trained facilitator directed the discussions, using a structured FGD guide. The FGD guide consisted of a brief explanation of the samples that were tasted during the sensory evaluation as well as a set of questions for initiating and facilitating the discussion. Guidelines for conducting focus group discussions with a structured set of open-ended questions were followed, as recommended elsewhere [44]. The question guide included questions that captured factors from themes that influence caregivers’ general acceptance of or willingness to use CFs. These themes included sensory attributes (taste, aroma, texture, and colour), physical access, affordability, cultural acceptability and feasibility of preparing a CF [1,45,46]. Table 3 shows the questions included in the FGD guide.

FGDs were facilitated in the local language (Luganda) by trained FGD facilitators. A digital voice recorder was used to record the FGDs after participants consented to the use of the voice recorder. The recordings were later translated from Luganda into English by the three FGD facilitators. The translated recordings were cross-checked by a Luganda professional teacher against the English translation, for accuracy.

### 2.5. Data Analysis

A sensory attribute was considered acceptable if it was rated as good to very good by caregiver panellists. The proportion of caregivers was calculated according to their sensory attribute ratings for the BPB and PB. A chi square test was conducted to test for significant differences in the sensory attributes (taste, colour, odour, texture and general acceptability) between BPB and PB at a P value of 0.05. Statistical and data analysis was achieved using STATA, version 13.1. 

Data generated from the FGDs was analysed using deductive thematic analysis. Thematic analysis is a method for identifying and analysing patterns (themes) of meaning in a dataset [47]. This study used a deductive thematic analysis because themes were predetermined before FGDs were conducted [48]. After conducting FGDs, themes were first summarised for each focus group, and then compared across all the seven FGDs to explore the most prominent themes, triangulate caregiver perspectives, and subsequently explore any potential context-specific variations associated with the predetermined themes of sensory attributes (taste, aroma, texture, and colour), physical access, affordability, cultural acceptability and feasibility of preparing a CF.

### 2.6. Ethical Approval

Permission to conduct the study was granted by the District Health Office, Kyankwanzi district, Uganda. In South Africa, Ethical approval was obtained from the Biomedical Research Ethical Committee, University of KwaZulu-Natal, South Africa (Reference number: BE 438/19). In Uganda, ethical approval was granted by The AIDS Support Organisation Research Ethical Committee (Reference number TASO-REC/066/19-UG-REC-009). Informed and signed consent were obtained individually from the caregivers who participated in the study.

## 3. Results

### 3.1. Background Characteristics of Caregivers 

A total of 70 eligible caregivers completed both the sensory evaluation study and FGDs. They included 63 (90%) and 7(10%) females and males, respectively. The mean age of the caregivers was 23.6 years. Only 24% of the caregivers had at least completed primary level education. The mean age of children belonging to the caregivers who participated in the study was 12.3 months. 

### 3.2. Sensory Evaluation 

Table 4 shows the sensory acceptability ratings for the BPB and PB, according to the different sensory attributes, number and percentage of caregivers who gave the different ratings for each sensory attribute of BPB and PB. 

The majority of the caregivers rated both CFs as good to very good. Seventy caregivers scored each sensory attribute of BPB as follows: 1(1.4%), 2(2.7%), 6(8.6%), 38(54.3%) and 23(32.9%) scored taste as very bad, bad, neutral, good and very good, respectively; 2(2.9%), 4(5.7%), 16(22.9), 16(22.9%) and 32(45.7%) scored texture as very bad, bad, neutral, good and very good, respectively; 3(4.3%), 3(4.3%), 12(17.1%), 28(40.0%) and 24(34.3%) scored aroma as very bad, bad, neutral, good and very good, respectively; 1(1.4%), 2(1.4%), 6(8.8%), 35(50%) and 27(38.6%) scored colour as very bad, bad, neutral, good and very good, respectively; 1(1.4%), 1(1.4), 2(2.9%), 36(51.4%), and 30(42.9%) scored overall acceptability as very bad, bad, neutral, good and very good, respectively. 

Furthermore, the 70 caregivers scored each sensory attribute of PB as follows: 1(1.4%), 0(0%), 2(2.9%), 47(67.1%) and 20(28.6%) scored the taste as very bad, bad, neutral, good and very good, respectively; 2(2.9%), 2(2.9%), 9(12.9), 38(54.3%) and 19(27.1%) scored texture as very bad, bad, neutral, good and very good, respectively; 2(2.9%), 2(2.9%), 9(12.9%), 38(54.3%) and 19(27.1%) scored aroma as very bad, bad, neutral, good and very good, respectively; 1(1.4%), 1(1.4%), 2(2.9%), 36(51.4%) and 30(42.9%) scored colour as very bad, bad, neutral, good and very good, respectively; 0(0%), 2(2.9%), 10(14.3%), 38(54.3%), and 20(28.6%) scored overall acceptability as very bad, bad, neutral, good and very good, respectively. 

### 3.3. Association of Sensory Acceptability between BPB and PB

A binary outcome of sensory acceptability (yes or no) was created for each sensory attribute of BPB and PB. The CF was regarded as unacceptable if caregivers scored the sensory attribute as very bad to neutral. In contrast, the CF was regarded acceptable if caregivers scored the sensory attributes as good to very good. Findings show that 64% to 96% of the caregivers rated the sensory attributes of both BPB and PB as acceptable (good to very good). Table 5 shows the association between the CFs and sensory acceptability. 

Out of the 70 caregivers, 61(87%), 48(69%), 52(74%), 62(89%), and 53(76%) scored taste, texture, aroma, colour and overall acceptability of BPB as acceptable (good to very good), respectively. Furthermore, out of the 70 caregivers, 67(96%), 45(64%), 57(81%), 66(94%), and 58(83%) scored taste, texture, aroma, colour and overall acceptability of PB as acceptable (good to very good), respectively. The chi square test revealed that there was no significant difference (P>0.05) in caregiver acceptability for all attributes between BPB and PB.

### 3.4. Focus Group Discussions 

#### 3.4.1. Taste

All participants in the focus group discussions indicated that they had tasted PB before but not BPB. They further noted that the taste of BP and BPB were similar. 

“I thought this was pumpkin alone (BPB). If you had not said that this is a mixture of pumpkin and common bean, I wouldn’t have realised that it was a mixture of the two food ingredients.” (Female caregiver).

Caregivers wondered why BPB and PB would taste the same, yet they had different ingredients. Therefore, most of the caregivers were interested to know the specific varieties used in the preparation of the BPB (male caregiver). 

“… how come that BP and BPB taste almost the same? We have several varieties of common bean such as *Obwayelo*, *Nambale*, *Obote*, *Kanyebwa* and several varieties of pumpkin such as *Bala*, *Sweet cream*, *Dulu*, *Ozinga*, *Wujju* among others. Could you please let us know the specific amounts of ingredients of pumpkin and common bean varieties used to prepare BPB?” (Female caregiver).

#### 3.4.2. Texture and Colour 

The softness and colour of the BPB appealed to caregivers. They perceived that children would also accept it. Caregivers were interested to know the ratio of pumpkin and common bean used to prepare the BPB. 

“The colour of BPB is not any different to the usual mashed pumpkin we give our children. This yellow colour is good for children since it is bright, infants and young children like colourful things.” (Female caregiver).

“In addition to the bright yellow colour, BPB was soft. Please what ratios of pumpkin and common bean did you use to prepare BPB?” (Female caregiver).

“As a matter of fact, we could not differentiate the softness between BPB and PB. It is important we know the ratios you used to mix pumpkin and common bean. This will help us to use these ratios when we are preparing BPB while at home.” (Female caregiver).

#### 3.4.3. Aroma

Caregivers indicated that the aroma of BPB was not any different from the PB.

“Recognising the difference in smell between BPB and PB was difficult. These two CFs smell the same.” (Male caregiver).

“For me I thought both foods were the same because they were almost similar in smell and colour.” (Female caregiver).

#### 3.4.4. Cultural Acceptability 

Caregivers agreed that the ingredients for BPB, i.e., pumpkin (*Sweet cream*) and common bean (*Obwelu*) are culturally acceptable for human consumption in their community.

“…on many occasions, our in-laws and husbands dictate on the new foods we have feed to our children. I remember, two years back, they introduced to us a nutritious maize porridge fortified with termites. However, my in-law and husband refused me to feed my twins with this porridge, because eating termites is not acceptable in their clan. BPB seems new to us. However, the common bean and pumpkin used to make BPB are culturally acceptable for consumption in our community. I am pretty sure that we shall not get any resistance from our in-laws and husbands to use them in the preparation of BPB.” (Female caregiver).

“Pumpkin and common bean are widely acceptable for consumption in Uganda and our community. As a matter of fact, common bean is frequently consumed in our households. If you need to confirm, just ask them, whether there is anyone who did not prepare common beans at their home in last two days….” (Male caregiver).

#### 3.4.5. Access and Affordability of Pumpkin and Common Bean

Caregivers revealed that pumpkin and common bean are easily accessible from their gardens or local markets and are affordable to them. However, they wanted to know the variety of pumpkin and common bean that was used to prepare the BPB so that they could cultivate or buy them for use in the preparation of BPB.

“Accessing common bean is never a problem to us. Almost every household cultivates common bean every season, and the surplus is sold off to traders.” (Male caregiver).

“For those who do not cultivate, common bean is affordable because on average, 1 kilogram of common bean costs 1000 Uganda shillings.” (Male caregiver). 1000 Uganda shillings is equivalent to 0.2 United States Dollars (USD). 

“Not very many households cultivate pumpkin as it is done with beans. However, the price of pumpkin is affordable at the local market. For example, on average a small to medium sized pumpkin costs 500 Uganda shillings.” (Male caregiver). 500 Uganda shillings is equivalent to 0.1USD.

“What specific variety of common bean and pumpkin did you use to prepare BPB? then we would cultivate them, because our main source of common bean is from our household gardens.” (Female caregiver).

#### 3.4.6. Feasibility to Prepare Common Bean and Pumpkin

Several caregivers emphasised that they would not frequently prepare common bean because it takes long to cook, hence consuming a lot of fuel. However, other caregivers advised their peers on how to reduce the cooking time of common bean. On the other hand, caregivers noted that pumpkin is easy to prepare because it cooks fast. 

“…cooking common beans takes quite a longer time, minimum of three hours. Ideally it consumes a lot of firewood or charcoal.” (Female caregiver).

“Now days firewood is very scarce, whilst charcoal is too expensive. As a matter of fact, to cook common bean, one must fill the charcoal stove three times before common beans are ready. This is unacceptably expensive to us.” (Female caregiver).

“Pumpkin is among the easiest foods to cook, once it starts boiling, it will be ready in a few minutes. However, common bean can take a couple of hours….” (Female caregiver).

“… reducing cooking time for common bean is possible by soaking them over night. Soaking makes common bean soft, and quick to get ready after boiling.” (Female caregiver).

“In addition, there is this type of salt called *Ekisula,* which also softens common bean when cooking, hence reducing cooking time.” (Female caregiver).

## 4. Discussion

Ugandan children are predominantly fed on CFs formulated from staple cereals and tubers, devoid of vitamin A, iron and zinc [10,11]. This increases child vulnerability to VAD, ID and ZnD. Therefore, the preparation of a novel BPB rich in PVAC, iron and zinc was necessary. Testing for caregiver acceptability of BPB was plausible because CFs disliked by caregivers are unlikely to be fed to children [30].

The results of this study indicate that the sensory attributes of BPB and PB were equally acceptable (rated as good to very good) to child caregivers who evaluated it. These findings are consistent with other studies conducted in Uganda and South Africa which revealed that micronutrient rich CFs prepared by home cooking methods were acceptable to child caregivers [45,46,49,50]. However, these studies formulated CFs similar to PB because they were predominantly rich in PVAC, as they used provitamin A biofortified foods such as orange fleshed sweet potato(OFSP) and provitamin A biofortified maize [45,46,49,50]. It is worth noting that this present study formulated a multiple micronutrient BPB rich in PVAC, iron and zinc. 

Based on Table 2, adding common bean to PB increased iron and zinc content from 0.57 mg and 0.23 mg in PB to 1.99mg and 1.8mg in BPB, respectively. In contrast, vitamin A reduced from 280.3 µgRAE in BP to 187 µgRAE in PB. The 2013 WHO/FAO Guidelines on formulated CFs for older infants and young children recommends that a CF should contribute at least 50% of the RDA [39]. The RDA for iron, zinc and vitamin A for a children 12 to 24 months old is 7 mg, 3 mg, and 300 µgRAE, respectively [37]. Therefore, the 50% of the RDA recommendation of a CF for a child 12 to 24 months would be 150 µg RAE, 3.5 mg and 1.5 mg of vitamin A, iron and zinc, respectively. Hence, PB would contribute 16%, 15% and 186% of iron, zinc and vitamin A, respectively of the 50% requirement of a CF for a child, 12 to 24 months old. In contrast, BPB would contribute 57%, 73% and 125% of iron, zinc and vitamin A of the 50% requirement of a CF for a child, 12 to 24 months old. To this end, BPB significantly contributes a higher proportion of iron and zinc content to meet child RDA. It is worth noting that both BPB and PB contributed over 100% vitamin A of the 50% RDA requirement of a CF for a child 12 to 24 months old. Therefore, BPB would be preferred to PB because it is a multiple micronutrient-rich CF compared to BP. Although there may be concern about the vitamin A toxicity or hypervitaminosis which may occur after consumption of either BPB or PB, vitamin A toxicity is not associated with PVAC intake, because the efficiency of PVAC absorption falls as PVAC intake increases [51].

Furthermore, during FGDs, caregivers had positive attitudes towards the taste, colour, texture and aroma of the BPB. They revealed that the taste, colour and aroma of BPB was the same as that of the regular PB they feed their children. This suggests that caregivers accepted the colour, aroma and taste of BPB because they were similar to that of the PB (control), hence the willingness to feed their children the BPB. Furthermore, caregivers noticed that the BPB was soft enough to prevent choking. This suggests that caregivers perceived the consistency/texture of the BPB to be suitable for their children in the age range of complementary feeding, and therefore was in accordance with the recommendations on consistency of homemade CFs [1]. 

Affordability and physical access to novel CFs are key factors that may influence its potential to be used in complementary feeding [1,45,46]. During FGDs, caregivers noted that they had adequate access to common bean and pumpkin from their own gardens. Moreover, those who could not access them from their gardens noted that the common bean and pumpkin from the local market was affordable to them. These findings confirm that pumpkin and common bean, the ingredients used to prepare PVAC with iron and zinc rich BPB, are locally available and affordable in Uganda [18,19,52]. 

It is worth noting that throughout the FGDs, caregivers were interested to know the specific varieties of pumpkin and common bean that were used in the preparation of the BPB. Furthermore, they wanted to know the ratios of common bean and pumpkin used to prepare the BPB. These findings suggest that caregivers were interested to know more in order to prepare the BPB in the future. Such caregiver interest indicates that there is a need for caregiver sensitisation to promote the BPB by providing adequate information, education and communication on its preparation. Moreover, conducting such sensitisations is highly recommended to improve complementary feeding practices [1]. Furthermore, the participation of caregivers in CF formulation and acceptability testing encourages them to gain and share nutrition knowledge and positive perceptions towards good feeding practices [53].

A long cooking time for common bean and the associated high cost of fuel were the main challenges that would prevent caregivers from preparing the BPB for children. However, peer caregivers indicated that soaking the common beans before cooking softens it, thus reducing the cooking time and use of fuel. The information shared by caregivers is consistent with a review of studies that demonstrated that common bean soaked before cooking, cooked faster compared to the non-soaked common bean [54]. It is worth noting that this study soaked common bean before cooking them during the preparation of the BPB. Sharing such nutrition related knowledge among expert and novice peer caregivers is necessary and recommended for promoting and supporting positive infant and young child feeding practices in the community [1]. 

Compared to other CF acceptability studies that recruited only female caregivers [46,50,55], this study recruited both male and female caregivers. It is worth noting that male involvement and participation in such complementary feeding studies is very important, particularly in Africa, where men/fathers are decision makers regarding how money is spent on food [56]. Moreover, male involvement is necessary in the promotion and support of adequate complementary feeding practices [57].

Furthermore, caregivers showed an interest in cultivating the pumpkin and common bean varieties used in the preparation of BPB. However, the yield and productivity of cultivated common bean and pumpkin in sub-Saharan Africa is influenced by agronomic factors such as soil fertility [58,59]. Accordingly, the district nutrition coordination committee, which is comprised of several food and nutrition security experts including agricultural extension workers, should support caregivers on how to improve soil fertility during the cultivation of common bean and pumpkin [60]. 

### Strengths and Limitations

The study analysed for micronutrients of public health importance in the formulated CFs [3]. However, it did not analyse for the percentage of moisture and calorie contents of the formulated CFs. It is worth noting that the calorie content is needed when calculating the nutrient density (ND) of a given food [61]. Nutrient density is a measure of the nutrients provided per calorie of food, or the ratio of nutrients to calories [61]. Since this study did not analyse the calorie content of the formulated CFs, it is difficult to calculate the ND for BPB and PB and compare it with other CF blends. 

Furthermore, PVACs are fat soluble, and therefore, incorporating fat during preparation of PVAC rich foods can increase PVAC bioavailability [62]. However, fat was not used during the preparation of BPB or PB. Although, the widely acceptable 2001 Institute of Medicine bioconversion recommendations of PVAC to retinol used in this this study are independent of the use of fat as an ingredient in the preparation of PVAC-rich foods [37], caregivers should be advised to add some fat when preparing or eating PVAC rich foods to increase vitamin A bioavailability [62]. 

## 5. Conclusions

A PVAC, iron and zinc rich complementary food, BPB, prepared from locally available pumpkin and common bean was acceptable to the child caregivers who tasted it. Caregivers were interested to know how to prepare and use it as a CF. These findings suggest that the BPB has the potential to be used by caregivers in complementary feeding to improve vitamin A, iron and zinc intake among children in the age range of complementary feeding, a group that is vulnerable to VAD, ID and ZnD. 

## Figures and Tables

**Table 1:** Ratio of mixing *Sweat cream* and *Obwelu* to formulate bean pumpkin blend

BPB Formulations	BPB -1	BPB-2	BPB-3
Ratio of mixing *Sweet cream* and *Obwelu*	1:1	1:2	2:1

BPB: Common Bean Pumpkin Blend.

**Table 2:** Micronutrient composition of edible portion of BPB and pumpkin blend (PB).

Micronutrient	BPB/100g	PB/100g	SCF^2^/100g
Iron (mg)	1.99	0.57	3.5
Zinc (mg)	1.08	0.23	1.5
β-carotene (µg)	2219	3326.5	
α-carotene (µg)	50.5	75.1	
Vitamin A (µg RAE)	187	280.3	150

BPB: Common Bean Pumpkin Blend; PB: Pumpkin Blend; RAE: Retinol Activity Equivalent (vitamin A). SCF^2^: Standard Complementary Food based on WHO/FAO (2013) recommendations [39], RAE= β-carotene (µg/100g)/12+ α-carotene (µg/100g)/24 [37]. BPB^1^ was prepared using 2 parts of pumpkin and 1 part of common bean.

**Table 3:** Focus group discussion data collection questions.

Focus Group Discussion Data Collection Questions
1. How would you grade the taste, colour, odour, and texture of the BPB compared to the PB?
2. To what extent is common bean and pumpkin accessible and affordable in your community?
3. How feasible would the preparation of common bean and pumpkin be during the development of BPB?
4. What cultural factors in your community or households may prevent you from using the BPB as a complementary food?

BPB: Common Bean Pumpkin Blend, PB: Pumpkin Blend.

**Table 4:** The number and percentage of caregivers who gave the different ratings for the sensory attributes evaluated for BPB and PB (*N* = 70).

CFS	Attributes	Very Bad n (%)	Bad n (%)	Neutral n (%)	Good n (%)	Very Good n (%)
BPB	Taste	1(1.4)	2(2.7)	6(8.6)	38(54.3)	23(32.9)
	Texture	2 (2.9)	4(5.7)	16(22.9)	16(22.9)	32(45.7)
	Aroma	3(4.3)	3(4.3)	12(17.1)	28(40.0)	24(34.3)
	Colour	1(1.4)	2(1.4)	6(8.8)	35(50)	27(38.6)
	Overall acceptability	1(1.4)	4(5.7)	12(17.1)	29(41.4)	24(34.3)
PB	Taste	1(1.4)	0(0)	2(2.9)	47(67.1)	20(28.6)
	Texture	2(2.9)	2(2.9)	13(18.6)	25(35.7)	20(28.6)
	Aroma	2(2.9)	2(2.9)	9(12.9)	38(54.3)	19(27.1)
	Colour	1(1.4)	1(1.4)	2(2.9)	36(51.4)	30(42.9)
	Overall acceptability	0(0)	2(2.9)	10(14.3)	38(54.3)	20(28.6)

CFS = Complementary Food Sample; BPB = Common Bean Pumpkin Blend; PB = Pumpkin Blend.

**Table 5:** Association of sensory acceptability between BPB and PB.

Sensory Attribute	Acceptable (N = 70 across rows)		Χ^2^	P Value
	Yes, n (%)	No, n (%)		
Tast			3.28	0.07
BPB PB	61(87) 67(96)	9(13) 3(4)		
Texture			0.29	0.59
BPB PB	48(69) 45(64)	22(31) 25(34)		
Aroma			1.11	0.31
BPB PB	52(74) 57(81)	18(26) 13(19)		
Colour			1.46	0.23
BPB PB	62(89) 66(94)	8(11) 4(6)		
Overall acceptability			1.09	0.30
BPB PB	53(76) 58(83)	17(24) 12(28)		

BPB: Bean Pumpkin Blend; PB: Pumpkin Blend, Χ^2^: Chi square test.
